# Bone Health in Space Flight: Incomplete Bone Mineral Density Convalescence at 1 Year Postmission Without Increased Fracture Risk

**DOI:** 10.5435/JAAOSGlobal-D-25-00155

**Published:** 2025-11-17

**Authors:** Benjamin Fiedler, Todd Phillips, Jad J. Lawand, Cameron Noorbakhsh, Abdullah N. Ghali, Jeffrey Hauck, Adil S. Ahmed

**Affiliations:** From the Department of Orthopedic Surgery, Baylor College of Medicine, Houston, TX (Dr. Fiedler, Dr. Ghali, and Dr. Adil); the Levy Shoulder to Hand Center at the Paley Orthopaedic Institute, FL (Dr. Phillips); the Department of Orthopaedic Surgery and Rehabilitation, University of Texas Medical Branch, Galveston, TX (Dr. Lawand); the Baylor College of Medicine, Houston, TX (Mr. Noorbakhsh and Mr. Hauck); and the Baylor College of Medicine Center for Space Medicine, Houston, TX (Dr. Adil).

## Abstract

**Introduction:**

Understanding the impact of space flight on orthopaedic health is crucial for optimization of astronaut health, space flight safety, and chance of mission success. This study sought to assess the rate and degree of bone mineral density (BMD) recovery across various anatomic regions on return to Earth, and further how the length of space flight and astronaut age affect BMD recovery.

**Methods:**

A retrospective cohort study was performed to quantify the changes in BMD and fracture risk after return to Earth. The Lifetime Surveillance of Astronaut Health epidemiology database at National Aeronautics and Space Administration provided preflight and postflight dual-energy radiograph absorptiometry data and Fracture Risk Assessment Tool scores for 94 astronauts. BMD loss and rate of recovery post-space flight was recorded and analyzed. Subanalyses were performed assessing effect of astronaut age and mission duration on BMD recovery and fracture risk.

**Results:**

In the hip and the spine, losses in BMD occurred that do not recover to preflight BMD levels by 1 year postflight. Astronauts who spent greater than 6 months in space flight recovered slower and more incompletely at the spine (*P* = 0.011), hip (*P* = 0.018), and femur (*P* = 0.049) compared with those who spent less than 6 months in space flight. No notable difference was observed in the risk of 10-year osteoporotic hip fracture based on duration of space flight or astronaut age.

**Conclusion:**

Increasing time in space flight leads to larger losses in BMD and slower BMD rate of return. At 1 year postflight, preflight BMD levels at the hip and spine are only achieved by 34.0% and 46.8% of astronauts, respectively.

The world has recently entered a new age of space exploration, pushing the boundaries of space flight to new and unprecedented levels. The advent of space tourism and commercial companies like Space X and Blue Origin has increased access to space flight to the general population.^1^ In addition, the National Aeronautics and Space Administration (NASA) is planning longer distance and longer duration missions than ever previously performed, such as missions to Mars.^2,3^ As access to space flight increases and aspirations of space flight change, understanding the effect of space flight on the musculoskeletal system has become paramount to ensure safety and health for all space travelers.

Space flight exposes astronauts to a uniquely challenging environment with notable orthopaedic implications.^4^ The human body is adapted to survive in a gravitational environment^5^; on Earth, the presence of a constant mechanical loading stimulus enables musculoskeletal metabolic homeostasis. In microgravity,^6^ the absence of this loading stimulus in conjunction with the effects of space radiation^7^ and vestibular dysfunction^8^ leads to notable physiologic changes highlighted by muscle atrophy, tendon laxity, loss of strength,^4^ and decreased bone mineral density (BMD), in addition to direct injury secondary to space flight related and extravehicular activity.

BMD is known to be especially affected by space flight.^9^ Previous studies demonstrated that astronauts experience a decrease in BMD of up to 0.5%-1.5% per month in space.^10,11^ Although implications of BMD reduction on Earth are well established, the impact of space flight-associated BMD loss is incompletely understood, as most research to date uses small cohorts and focuses on short-term outcomes.^12^ As a result, many questions remain surrounding long-term recovery of BMD on return to gravitational environment. Therefore, this study sought to assess the rate and degree of BMD recovery across various anatomic regions on return to Earth and further how length of space flight and astronaut age affect BMD recovery. We hypothesized that BMD would markedly decrease in space flight and recovery of axial skeleton BMD will be incomplete 1 year post-space flight. However, owing to astronaut age and health, the reductions in BMD will not affect Fracture Risk Assessment Tool (FRAX) scores.

## Methods

A retrospective cohort study was conducted to compare and quantify the changes in BMD after return to Earth among astronauts based on (1) the length of space flight missions, and (2) the age of the astronauts was developed following approval by the Institutional Review Board. A total of 94 astronauts met inclusion criteria. Of the 94 astronauts 31 were older than 45 years and 39 flew on missions for greater than 6 months. The Lifetime Surveillance of Astronaut Health (LSAH) epidemiology database at NASA was queried to provide dual-energy radiograph absorptiometry (DEXA) data of astronauts for preflight, 5-day return, 6-month return, 1-year return, 2-year return, and 3-year return from space flight data. Dual-energy radiograph absorptiometry scan values were provided for the forearm, spine, greater trochanter, femoral neck, and hip, which includes BMD of the entire proximal femur.^13^ Inclusion criteria included all persons who participated in space flight with at least one return from space flight DEXA scan. Astronauts without preflight DEXA scans were excluded. National Aeronautics and Space Administration epidemiologists reviewed and compiled the astronaut medical records.

Owing to safety and governmental regulated data sharing restrictions, astronaut epidemiologic information was parsed into dichotomous categories by NASA LSAH before the study team was given access. Subjects were categorized by age older than or younger than 45 years at time of space flight and length of space flight mission greater than or less than six months. To maintain appropriate deidentification of data, NASA LSAH provided only DEXA scan value outputs for both BMD and their correlative T scores. Similarly, all FRAX score calculations were performed by NASA LSAH with each astronaut's individual risk factors per the FRAX calculator (https://www.fraxplus.org/calculation-tool) and only composite scores were received by the authors.

## Statistical Analysis

With the available data, patients were separated into cohorts by age—older than 45 years and younger than 45 years—as well as by duration of space travel—greater than 6 months and less than 6 months. The relative decrease in DEXA T scores were compared between these cohorts. A subsequent analysis was performed on the rate of recovery of BMD after return from space flight. Given the drop-off of data at the 2-year and 3-year postmission marks (∼70% missing), only the 5-day, 6-month, and 1-year time points were used for this analysis. For each astronaut, the 5-day, 6-month, and 1-year T scores were plotted, and slopes of each cohort, representing pace of recovery, were compared using *t*-test for regression. Finally, descriptive statistics were used to analyze differences in FRAX score for each cohort at 1-year postmission. The Student *t*-test was used to determine differences in continuous variables. Chi square was used to determine differences in categorical variables. Were the assumptive conditions of the chi-square not met, the Fisher exact test was used to determine statistical differences. Alpha was set to significance level of 0.05 for all analyses. Statistical analyses were performed with IBM SPSS Statistics V.27.0 (IBM) and R software 3.6.1 (www.r-project.org).

## Results

A total of 94 astronauts met inclusion criteria. Table 1 outlines the separation of astronauts into cohorts by age and duration of space flight.

**Table 1:** Astronaut Age and Flight Duration

Demographics	
N (%)	94
Age	
<45 yrs	63 (67%)
>45 yrs	31 (33%)
Space flight	
<6 mo	55 (58.5%)
>6 mo	39 (41.5%)

### Changes in Bone Mineral Density from Space Flight

**Figure 1:**
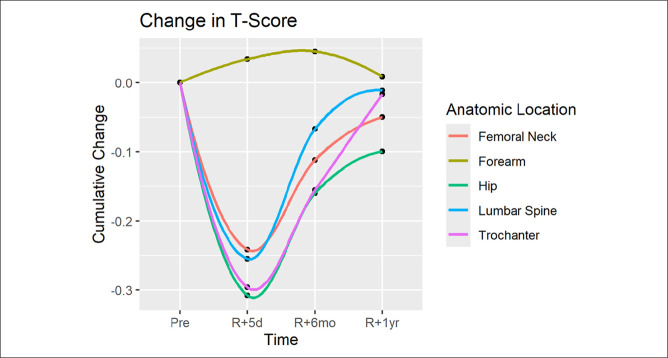
Graph showing cumulative changes in dual-energy radiograph absorptiometry T score based on anatomic location.

Figure 1 outlines the average changes in BMD at each anatomic region—forearm, lumbar spine, femoral neck, greater trochanter, and hip—based on DEXA T scores at preflight, 5-day, 6-month, and 1 year postmission data. In the lower extremity and spine, the overall trend showed a stark decrease in T score because of space flight, with a slow recovery over 1 year that did not reach preflight BMD. Conversely, in the forearm, the trend showed slight increase in BMD with a gradual return to preflight normal over the year.

### Bone Mineral Density Decline Preflight Versus 5 Days Postmission

The average decline in BMD of all 94 astronauts after space flight was 0.03 for the forearm, 0.28 for the spine, 0.27 for the femoral neck, 0.30 for the greater trochanter, and 0.31 for the hip.

**Figure 2:**
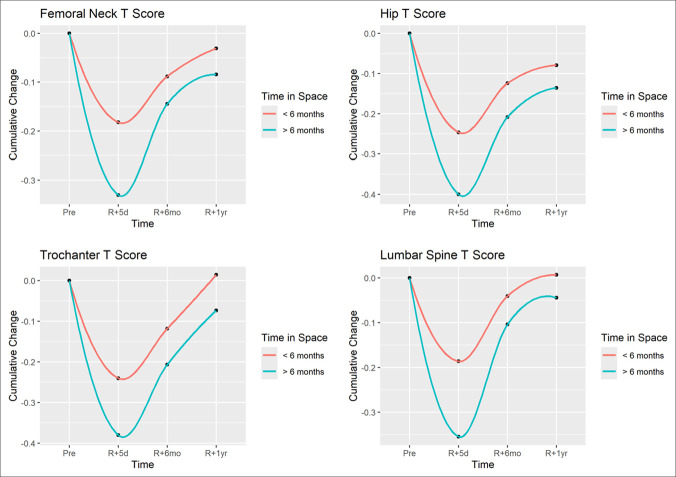
Graph showing comparison of rate of recovery of bone mineral density at 1 year with preflight values based on space flight duration—greater than 6 months vs. less than 6 months. **A**, Femoral neck dual-energy radiograph absorptiometry T score, (**B**) Hip dual-energy radiograph absorptiometry T score, (**C**) Greater trochanter dual-energy radiograph absorptiometry T score, and (**D**) Lumbar spine dual-energy radiograph absorptiometry T score.

When stratified for space flight duration, the average change in preflight to 5-day return BMD was significantly different between the astronaut cohorts greater than 6 months and less than 6 months for the hip (0.45 vs. 0.21, *P* < 0.001), femoral neck (0.41 vs. 0.18, *P* < 0.001), greater trochanter (0.46 vs. 0.20, *P* < 0.001), and spine (0.42 vs. 0.19, *P* = 0.006). These results are portrayed in Figure 2. However, the forearm was not significantly different (−0.04 vs. −0.03, *P* = 0.422).

**Figure 3:**
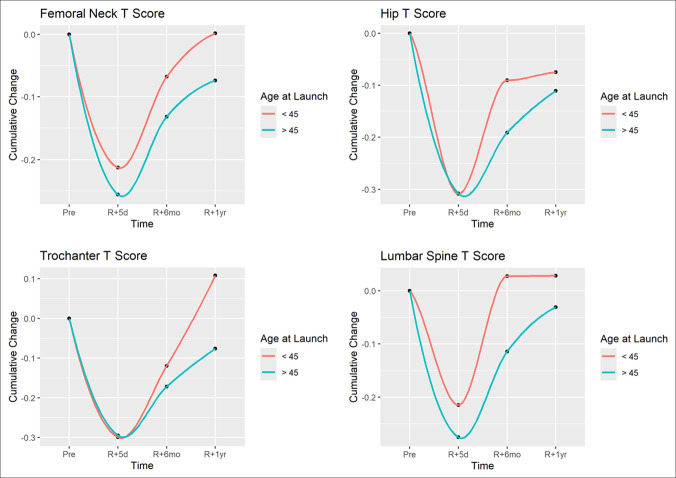
CGraph showing comparison of rate of recovery of bone mineral density at 1 year with preflight values based on astronaut age—older than 45 years versus younger than 45 years. **A**, Femoral neck dual-energy radiograph absorptiometry T score, (**B**) Hip dual-energy radiograph absorptiometry T score, (**C**) Greater trochanter dual-energy radiograph absorptiometry T score, and (**D**) Lumbar spine dual-energy radiograph absorptiometry T-score.

When stratified by astronaut age, the average change in preflight to 5-day return BMD was not significantly different between the older than 45 and younger than 45 cohorts for the hip (*P* = 0.377), femoral neck (*P* = 0.580), greater trochanter (*P* = 0.434), spine (*P* = 0.517), or the forearm (*P* = 0.092). These results are portrayed in Figure 3.

### Bone Mineral Density Recovery Postmission

When assessing recovery of BMD on return to gravitational environment, the greater than 6-month space flight cohort recovered significantly slower than astronauts who flew less than 6 months for the spine (*P* = 0.011), hip (*P* = 0.018), and femur (*P* = 0.049; Figure 2). No notable difference was observed in recovery rate at the forearm and greater trochanter. When stratified by age, no notable difference was observed in recovery rate in the forearm, spine, femoral neck, greater trochanter, or hip (Figure 3).

### Fracture Risk Assessment Tool Analysis

Given the changes in BMD, an analysis of 10-year fracture risk for an osteoporotic hip fracture was used with the FRAX scoring tool (https://www.fraxplus.org/calculation-tool) to assess the long-term effects of changes to astronaut BMD. Preflight FRAX score was 0.09%, ranging from 0 to 0.50%. Upon return at 1 year, the average FRAX score was 0.11%, ranging from 0% to 0.80%. No notable difference was observed in the risk of 10-year osteoporotic hip fracture based on duration of space travel, with 0.09% at preflight to 0.114% at 1 year in the greater than 6 months cohort and 0.09% preflight and 0.12% at 1 year postmission in the less than 6 months cohort. Although there was a larger difference based on astronaut age, there was no notable difference in 10-year osteoporotic hip fracture risk with 0.11% at preflight to 0.13% at 1 year in the older than 45 cohort, and 0.06% preflight and 0.08% at 1 year postmission in the younger than 45 cohort.

## Discussion

The primary objective of this study was to compare the changes in DEXA measured BMD T scores based on time in space flight (greater than or less than 6 months) and age (older than or younger than 45 years). Secondary objectives of this study were aimed at modeling the return of BMD T scores to preflight levels over the first year of return, comparing anatomic differences in changes to BMD with space travel, and comparing FRAX scores between cohorts. The findings of this study confirm the hypothesis; most significantly this analysis of 94 astronauts showed that increased duration in space flight increased the precipitous declination in BMD T scores secondary to the microgravity environment and slowed the rate of return to pre-flight BMD *t-*score level.

For all astronauts, BMD T scores on initial return from space flight were markedly decreased compared with preflight scores in the hip, femur, greater trochanter, and spine (Figure 1). Notably, the cohort spending more than 6 months in space experienced greater reductions in initial DEXA testing. There was more than a twofold declination in BMDT scores at both the hip (0.45 for >6 months vs. 0.21 for <6 months, *P* < 0.001) and spine (0.42 for >6 months vs. 0.19 for <6 months, *P* = 0.006). Dual-energy radiograph absorptiometry measurements of astronauts dating back decades have demonstrated 0.5% to 1.5% reductions in BMD per month of time spent in space flight,^11,14^ which is consistent with the decline seen in this study. Owing to data stratification by NASA LSAH, regression analysis comparing BMD t-score changes to specific flight times could not be performed in this study; however, recent meta-analyses have shown that this loss tends to occur at a near linear rate.^12,15^ As a result, astronauts who spend more time in space flight are likely to experience greater reduction in BMD directly proportional to the length of time spent in space.

On return from space flight, rate of BMD restoration was initially substantial for most astronauts. However, the rate of return appeared to progressively taper and plateau as time from space flight return increased with some astronauts experiencing especially slow and limited recovery, often failing to reach preflight levels at 1 year postflight (Figure 1). When stratifying by space flight time, the greater than 6-month cohort had significantly slower rate of recovery in the hip (*P* = 0.018) and spine (*P* = 0.011) over the first year upon return to gravity when compared with the less than 6-month cohort. Although research into long-term recovery of BMD post-space flight is limited, Gabel et al^16^ analyzed DEXA results at the distal tibia in astronauts pre- and post-space flight and found that tibial BMD remained approximately 1% to 2% below pre-flight levels at 1 year follow-up, accordant to the current study results. Moreover, Gabel et al^16^ demonstrated that astronauts on longer missions displayed less complete bone recovery,^16^ mirroring the outcomes seen in the greater than 6-month space flight time cohort. Similarly, Coulombe et al^9^ analyzed vertebral changes from preflight to 1 year postflight and found that BMD remained approximately 4.66% below preflight levels, congruent with our study findings. Both the Gabel et al and Coulombe et al studies were limited by smaller sample sizes because of difficulty in obtaining astronaut data; however, our study is the largest analysis of BMD among astronauts and is in concordance with previous literature.

Differences in the rate of post-space flight BMD recovery compared with intra-space flight loss highlights the importance of persistently decreased DEXA measurements at the hip and spine at 1 year post-space flight. Unlike space flight-associated bone density loss which occurs at a linear rate, recovery occurs at a logarithmic rate,^17^ with a rapid initial rate of BMD recovery upon re-exposure to gravitational environment, which gradually plateaus. In our study, the greater than 6-month cohort experienced nearly four times as much BMD recovery from immediate postflight to 6 months postflight as they did from 6 months postflight to 1 year postflight, highlighting the diminishing returns of the logarithmic recovery over time (Figures 1–3). In addition, there is incomplete recovery of BMD T scores at 1 year postflight with 66.0% incomplete recovery at the hip and 53.2% incomplete recovery in the spine. As a result, BMD recovery at time points after 1 year space flight is likely to occur at an increasingly slower rate and may result in permanently incomplete recovery. Subsequently, many astronauts often live extended periods with BMD levels below their preflight levels, and some astronauts may never return to preflight levels. Although most astronauts remain at levels that do not qualify as osteoporotic, five astronauts in this study experienced bone density reductions that newly classified them as osteopenic, which has further implications for this population with increasing age in regard to mid- and long-term fracture risk.

Notably, although BMD of the axial skeleton seems to decrease then gradually increase on return from space flight, BMD of the forearm seems to demonstrate the opposite trend, increasing during space flight then gradually decreasing on return. As the absence of a gravitational stimulus prevents astronauts from partaking in typical bipedal ambulation as they do on Earth, they must rely on upper extremity propulsion based mobility, inherently becoming functional quadrupeds. As a result, astronaut upper extremities are subjected to greater loading forces in space flight compared with on Earth, which may be contributing to this effect.

Despite the effects of space travel on bone density measurements demonstrated in this study, fracture risk assessment (FRAX) analysis demonstrated the 10-year fragility fracture risk in astronauts was not markedly different between the space flight cohorts. However, one limitation of FRAX analysis in this population is patient age. Ten-year fragility fracture risk increases nonlinearly as age increases when transitioning from an osteopenic state to an osteoporotic state, with an inflection point around age 65 years.^18,19^ The average age of astronauts in this study was 48 years, and as a result, this younger age of astronauts in this study likely heavily contributed to the analysis findings. However, in an older population or in those with already low preflight bone density, the impact of space flight on mid- to long-term fracture risk may be more notable.

The implications of this study are broad and numerous. To date, the most current terrestrial correlative is related to disuse osteopenia. However, as access to space flight increases with the development of the space tourism and commercial industry, these findings may become increasingly applicable and generalizable to clinical practice especially in high-risk individuals, such as elderly individuals or those with medical pathology tied to lower bone density values. These patients may experience marked reductions in bone density with space flight, posing exponential fracture risk; thus, musculoskeletal health guidelines or prophylaxis for private space flight may become a worthwhile future consideration. Furthermore, as plans for longer distance and longer duration missions, like missions to Mars, become increasingly plausible, one must consider risks associated with intramission and postmission fracture. To date, no fractures have been reported in space flight. However, risk may be introduced on exposure to partial gravity environments, like Mars, whose gravitational environment is approximately one-third than that of Earth. Notable reductions in BMD on landing in a partial gravitational environment like Mars, combined with need for immediate strenuous activity, like habitat building, pose serious hazards to astronauts as fractures in this environment would incur tremendous morbidity and mortality risks. Moreover, missions to Mars will subject astronauts to markedly longer periods of microgravity than ever previously experienced. BMD models predict astronauts will lose 32.4% to 36.8% of their femoral neck density and return to Earth with T scores of −1.49 to −2.93, well within the osteopenic to osteoporotic range.^20^ The results of this study suggest that in these long-duration mission astronauts will return with severely diminished BMD T scores. These will further likely not fully recover to preflight levels over the course of a year, subjecting space travelers to acutely high fragility fracture risk.

This study is not without limitations. Most importantly to maintain confidentiality of astronaut information, data were stratified with broad categorizations to classify space flight as “short” or “long.” Although 6 months was determined to be an appropriate cutoff by NASA LSAH, it is possible that bone density related risks change at periods other than 6 months. Demographic data related to sex and race were also not accessible in this study because of NASA LSAH confidentiality. This was partially mitigated through use of DEXA BMD T scores, which inherently rely on sex and race information to calculate. Moreover, it is unknown whether the same DEXA scan machines were used for each astronaut or at each time point for a singular astronaut, as each machine can be calibrated differently subjecting the outputs to an assumed standard of error.^21^ Furthermore, this study was designed to assess patterns in BMD changes and does not report clinical outcomes or patient-reported outcomes. The addition of these data would provide further context to better understand implications of the reported changes in bone density. Moreover, astronauts may have been subjected to different recovery protocols upon return to Earth which could affect BMD recovery. In addition, there are inherent changes in BMD over the course of life regardless of space flight exposure, and these changes could be reflected in the data. Finally, only 20% astronauts in this study obtained BMD measurements beyond 1 year postflight; thus 2- and 3-year data were not included. Trending BMD recovery for longer periods may provide additional insight into BMD recovery in the long term.

## Conclusion

Increased space flight time leads to larger loss in BMD and slower rate of return to preflight BMD T score levels. At 1 year postflight, preflight BMD levels at the hip and spine are only achieved by 34.0% and 46.8% of astronauts, respectively. Musculoskeletal health guidelines for space flight may be a worthwhile consideration to mitigate these findings.

## References

[1] Krittanawong C Singh NK Scheuring RA : Human health during space travel: State-of-the-art review. Cells 2022;12:40. 36611835 10.3390/cells12010040 PMC9818606

[2] Siegel B Spry JA Broyan J : Development of a NASA roadmap for planetary protection to prepare for the first human missions to Mars. Life Sci Space Res (Amst) 2023;38:1-7. 37481303 10.1016/j.lssr.2023.03.009

[3] NASA's Human Path to Mars—NASA Science. 2014. https://science.nasa.gov/resource/nasas-human-path-to-mars/

[4] Lee Satcher R Fiedler B Ghali A Dirschl DR: Effect of spaceflight and microgravity on the musculoskeletal system: A review. J Am Acad Orthop Surg 2024;32:535-541. 38652883 10.5435/JAAOS-D-23-00954

[5] Adamopoulos K Koutsouris D Zaravinos A Lambrou GI: Gravitational influence on human living systems and the evolution of species on Earth. Molecules 2021;26:2784. 34066886 10.3390/molecules26092784 PMC8125950

[6] Man J Graham T Squires-Donelly G Laslett AL: The effects of microgravity on bone structure and function. NPJ Microgravity 2022;8:9. 35383182 10.1038/s41526-022-00194-8 PMC8983659

[7] Willey JS Lloyd SAJ Nelson GA Bateman TA: Space radiation and bone loss. Gravit Space Biol Bull 2011;25:14-21. 22826632 PMC3401484

[8] Vignaux G Ndong JD Perrien DS Elefteriou F: Inner ear vestibular signals regulate bone remodeling via the sympathetic nervous system. J Bone Miner Res 2015;30:1103-1111. 25491117 10.1002/jbmr.2426 PMC4772960

[9] Coulombe JC Senwar B Ferguson VL: Spaceflight-induced bone tissue changes that affect bone quality and increase fracture risk. Curr Osteoporos Rep 2020;18:1-12. 31897866 10.1007/s11914-019-00540-y

[10] Orwoll ES Adler RA Amin S : Skeletal health in long-duration astronauts: Nature, assessment, and management recommendations from the NASA bone summit. J Bone Miner Res 2013;28:1243-1255. 23553962 10.1002/jbmr.1948

[11] Juhl OJ Buettmann EG Friedman MA DeNapoli RC Hoppock GA Donahue HJ: Update on the effects of microgravity on the musculoskeletal system. NPJ Microgravity 2021;7:28. 34301942 10.1038/s41526-021-00158-4 PMC8302614

[12] Stavnichuk M Mikolajewicz N Corlett T Morris M Komarova SV: A systematic review and meta-analysis of bone loss in space travelers. NPJ Microgravity 2020;6:13. 32411816 10.1038/s41526-020-0103-2 PMC7200725

[13] Leslie WD Lix LM Tsang JF Caetano PA; Manitoba Bone Density Program: Single-site vs multisite bone density measurement for fracture prediction. Arch Intern Med 2007;167:1641-1647. 17698687 10.1001/archinte.167.15.1641

[14] LeBlanc A Schneider V Shackelford L : Bone mineral and lean tissue loss after long duration space flight. J Musculoskelet Neuronal Interact 2000;1:157-160. 15758512

[15] LeBlanc A Lin C Shackelford L Muscle volume, MRI relaxation times (T2), and body composition after spaceflight. J Appl Physiol 2000;89:2158-2164. 11090562 10.1152/jappl.2000.89.6.2158

[16] Gabel L Liphardt AM Hulme PA : Incomplete recovery of bone strength and trabecular microarchitecture at the distal tibia 1 year after return from long duration spaceflight. Sci Rep 2022;12:9446. 35773442 10.1038/s41598-022-13461-1 PMC9247070

[17] Sibonga JD Evans HJ Sung HG : Recovery of spaceflight-induced bone loss: Bone mineral density after long-duration missions as fitted with an exponential function. Bone 2007;41:973-978. 17931994 10.1016/j.bone.2007.08.022

[18] Tomasevic-Todorovic S Vazic A Issaka A Hanna F: Comparative assessment of fracture risk among osteoporosis and osteopenia patients: A cross-sectional study. Open Access Rheumatol Res Rev 2018;10:61-66. 10.2147/OARRR.S151307 PMC5985792 29881314

[19] Unnanuntana A Gladnick BP Donnelly E Lane JM: The assessment of fracture risk. J Bone Joint Surg Am 2010;92:743-753. 20194335 10.2106/JBJS.I.00919 PMC2827823

[20] Axpe E Chan D Abegaz MF : A human mission to mars: Predicting the bone mineral density loss of astronauts. PLoS One 2020;15:e0226434. 31967993 10.1371/journal.pone.0226434 PMC6975633

[21] Lee K Al Jumaily K Lin M Siminoski K Ye C: Dual-energy x-ray absorptiometry scanner mismatch in follow-up bone mineral density testing. Osteoporos Int 2022;33:1981-1988. 35614236 10.1007/s00198-022-06438-3

